# Global research trends on the links between gut microbiota and cancer immunotherapy: A bibliometric analysis (2012-2021)

**DOI:** 10.3389/fimmu.2022.952546

**Published:** 2022-08-24

**Authors:** Shanshan Yang, Suya Zhao, Yixiang Ye, Liqun Jia, Yanni Lou

**Affiliations:** ^1^ Graduate School, Beijing University of Chinese Medicine, Beijing, China; ^2^ Oncology Department of Integrated Traditional Chinese and Western Medicine, China-Japan Friendship Hospital, Beijing, China

**Keywords:** immunotherapy, gut microbiota, cancer, research trends, highly cited papers, bibliometrics

## Abstract

**Background:**

There is a crosstalk between gut microbiota (GM) and cancer immunotherapy (CI). The purpose of this study is to use bibliometric analysis to identify the highly cited papers relating to GM/CI and explore the research status and development trends of the GM/CI research.

**Methods:**

A literature search regarding GM/CI publications from 2012 to 2021 was undertaken on July 4, 2022. The article titles, journals, authors, institutions, countries, total citations, keywords, and other information were extracted from the Science Citation Index Expanded (SCIE) of Web of Science Core Collection (WoSCC). The Bibliometrix of R package and VOSviewer were used for bibliometric analysis.

**Results:**

A total of 665 papers were extracted. The number of papers has increased rapidly over the past decade, especially after 2018. The United States and China had the most publications and made great contributions to this field. Th5e Univ Texas MD Anderson Canc Ctr and Univ Paris Saclay were absolutely in the leading position in GM/CI. The most influential authors were Zitvogel L and Routy B. *Frontiers in Immunology* had the most publications and *Science* had the most total citations. Historical direct citation analysis explained the historical evolution in GM/CI. Highly cited papers and high-frequency keywords illustrated the current status and trends of GM/CI. Four clusters were identified and the important topics included the role of GM and antibiotics in CI, the methods of targeting GM to improve CI outcomes, the mechanism by which GM affects CI and the application of ICIs in melanoma. “Tumor microbiome”, “proton pump inhibitors” and “prognosis” may be the new focus of attention in the next few years.

**Conclusion:**

This study filtered global publications on GM/CI correlation and analyzed their bibliometric characteristics, identified the most cited papers in GM/CI, and gained insight into the status, hotspots and trends of global GM/CI research, which may inform researchers and practitioners of future directions.

## Introduction

In the last decade, cancer immunotherapy (CI) represented by immune checkpoint inhibitors (ICIs) has revolutionized clinical practice in oncology as an emerging therapeutic approach and ushered in a new era of cancer treatment (1–3). Immunotherapy has been shown to be effective in oncology treatment (4, 5), whether used alone or in combination with other anti-tumor methods (6, 7), significantly improving the health-related quality of life in cancer patients. However, its efficacy is still limited by the heterogeneity of the patients’ immune response and the heterogeneity among different tumors (8, 9), and some patients will experience primary or acquired drug resistance and related adverse events (10, 11) during treatment, which greatly hinder the widespread clinical application of anti-tumor immunotherapy.

Gut microbiome (GM) plays a key regulatory role in cancer development and treatment, as well as the immune response of the body, influencing anti-tumor immunosurveillance (12, 13). Preclinical and clinical studies in recent years also have found that GM can modulate anti-tumor immune response and affect the efficacy and toxicity of CI, especially ICIs (14, 15). In addition, GM can be involved in inducing inflammation or indirectly participating in cancer treatment through derived metabolites, ultimately affecting anti-tumor therapeutic effects, and can also be used as one of the biomarkers to predict tumor patients’ response to immunotherapy and potential prognosis (16). There is an intricate crosstalk among the GM, cancer immune response and immunotherapy (17).

Bibliometric analysis is a method of statistically evaluating the research status and trends, as well as the most influential studies in a specific field, and has been successfully applied to relevant research in medicine (18, 19). Citation analysis is one of the main methods of bibliometrics, which can evaluate the quality and recognition of articles, and better understand the discipline construction and development of a research field. At present, many immunotherapy-related areas have been well researched and explored through bibliometric analysis, such as the analyses of highly cited articles in programmed cell death protein 1 (PD-1) and programmed cell death ligand 1(PD-L1) immunotherapy (20), immunotherapy for hepatocellular carcinoma (21) and colorectal cancer (22), and emerging chimeric antigen receptor-based immunotherapy (23). However, currently, there is no published paper on the quantitative analysis of interactions between GM and CI. Therefore, this article aims to review the regulatory role of GM in CI especially ICIs immunotherapy in recent years, identify the related articles in GM/CI and analyze their characteristics, and look forward to providing references for researchers in the field of GM/CI.

## Materials and methods

### Data source and search strategy

Web of Science (http://www.webofknowledge.com) is an important database platform for obtaining global academic information. It includes a variety of influential international academic journals in the world, covering natural science, social science, art and humanities and other disciplines. In addition, Web of Science has a strict screening mechanism, which only includes important academic journals in various disciplines according to Bradford’s law in bibliometrics. Science Citation Index Expanded (SCIE) of Web of Science Core Collection (WoSCC) includes the most authoritative and influential mainstream academic journals in the field of natural science. Therefore, we chose SCIE of WoSCC as the search source.

**Figure 1:**
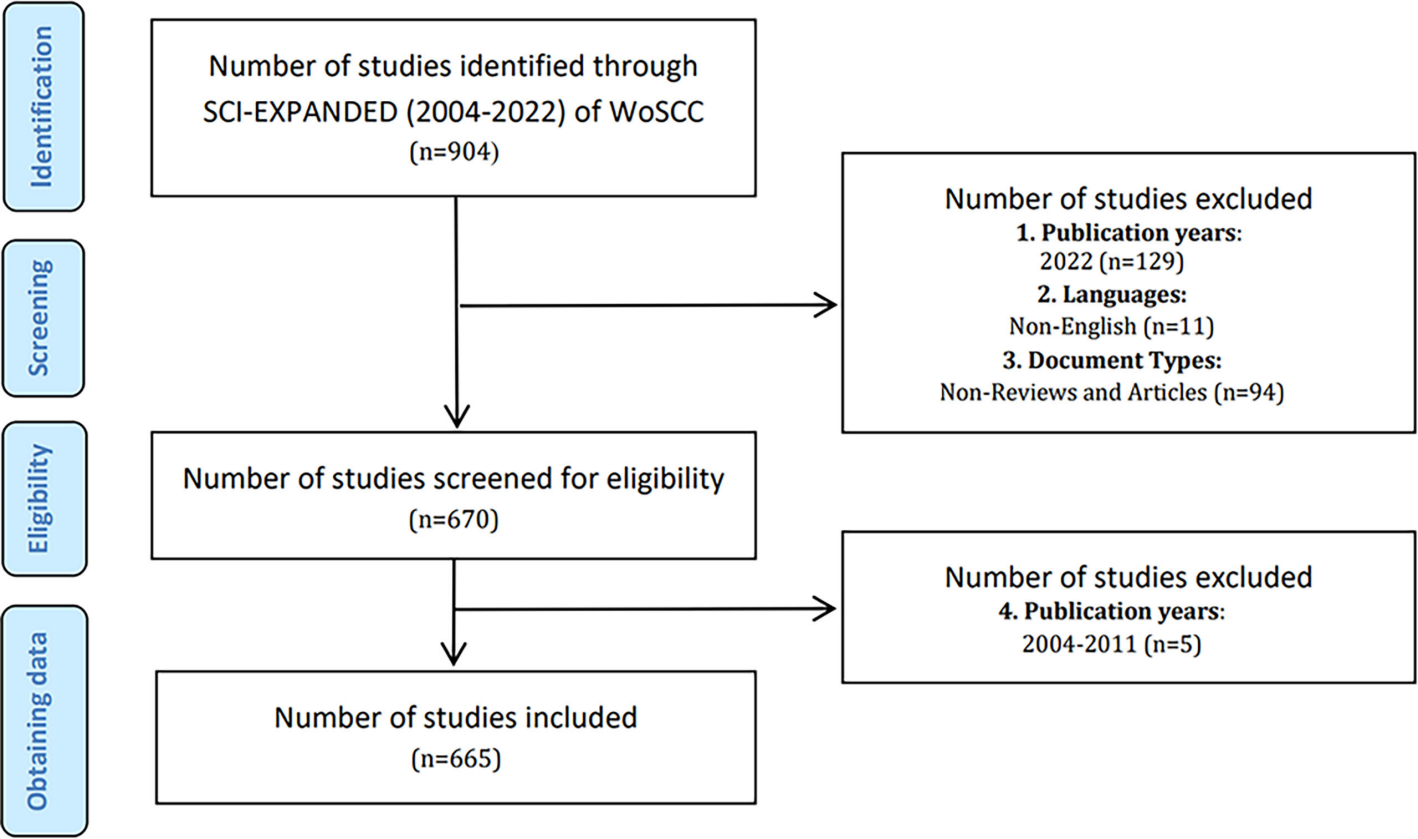
Flow diagram of literature search and screening in GM/CI.

All searches were performed on the same day (July 4, 2022) to avoid the bias caused by database updates. The data were retrieved from the SCIE of WoSCC database on July 4, 2022. Using the subject term “advanced search” method, the search terms were TS = “immunotherapy” and “gut microbiota” and “cancer” and their synonyms. Terms related to immunotherapy, gut microbiome and cancer entered into the WoS engine were extracted from the Medical Subject Headings (MeSH) in PubMed, and the wildcard “*” was used in place of any number of characters for the most comprehensive search of relevant literature. The detailed search strategy is in **Supplementary Material S1**. The selection criteria were as follows: (1) The period of the literature search was from January 1, 2012, to December 31, 2021, (2) Document types were limited to “article” and “review”, (3) The language type was set to only English. After screening, a total of 665 papers were finally obtained (**Figure 1**), of which 294 were “articles” and 371 were “reviews”. Two researchers (SY and SZ) independently performed the search and data extraction. We extracted all available information such as title, author, institution, country, publication year, and keywords from the raw data and exported records to the plain text file.

### Data analysis and parameter query

Bibliometric analysis was performed using a specific program from Rstudio’s Bibliometrix R package (version 2022.03.10, RStudio team, Boston, MA, USA), VOSviewer (version 1.6.18, Leiden University Science and Technology Research Center, the Netherlands) and Microsoft Excel 2019 (Microsoft, Redmond, Washington, USA). VOSviewer is a software developed for building and visualizing bibliometric networks. The Bibliometrix (https://www.bibliometrix.org) is an open-source tool for quantitative research in scientometrics. Each software allows for the construction and visualization of bibliometric networks to facilitate understanding of the GM/CI research. Specifically, the distribution of each component analyzed in the bibliometric analysis was assessed by a software package applying machine learning. For this, we used the following variables: annual scientific production, average citations per year, most relevant journals, journals dynamics, most impact journals by H-index or total citations (TC), top journals’ production over time, most relevant authors, top authors’ production over time, author local impact, most relevant affiliations, relevant funding agencies, country scientific production, collaboration network of countries, corresponding author’s country, top countries’ production over time, historical direct citation network, most global cited papers, most relevant keywords and cluster analysis of keywords. The journals’ impact factor (IF) and partition refer to the “2020 Journal Citation Reports”.

## Results

### Distribution of annual documents in GM/CI research

#### Annual development trend of publications

**Figure 2:**
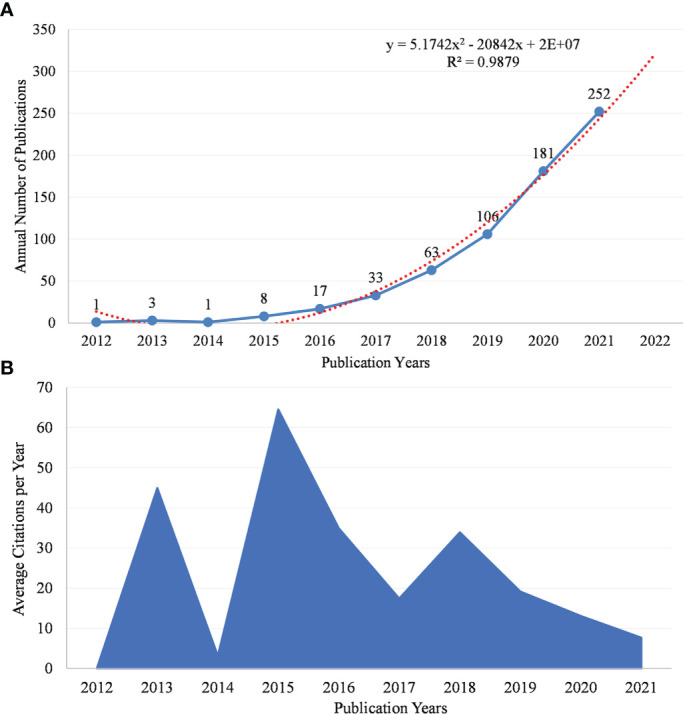
**(A)** Annual scientific production and the polynomial curve fitting of publications growth in GM/CI. **(B)** The number of average citations per year in GM/CI.

Analyzing the distribution of published papers from time series can reflect the trend of research. **Figure 2A** shows the number of GM/CI research papers from 2012 to 2021. As we can see, the number of papers (Np) was relatively small in 2012-2014 (n = 5, 0.75%) and increased slowly in 2015-2017 (n = 58, 8.72%). The Np increased rapidly between 2018 and 2021 (n = 602, 90.53%). A polynomial regression model *(f(x)=p_0_x^n^+p_1_x^n−1^+p_2_x^n−2^+p_3_x^n−3^+…+p_n_)* was constructed by Microsoft Office Excel 2019 to predict the Np published in 2022. By fitting data, a time prediction curve model was constructed and the formula was y=5.1742x^2^-20842x+2E+07. We observed a statistically significant relationship between the year and the number of publications (R² = 0.9879), and the fitting degree was good. According to the fitting curve, we estimated that the number of publications will reach about 320 in 2022.

#### Annual development trend of citations

The average annual citations of articles showed dynamic changes, as evidenced by the average citation distribution of papers per year (**Figure 2B**). The years with the highest average annual citations were 2013, 2015 and 2018, about 45, 65 and 34 times (the three peaks in **Figure 2B**), respectively, indicating that the papers from these three years had important research significance. As shown in **Table 1**, the respective highly cited papers of Iida N (2013), Sivan A (2015), Vetizou M (2015), Routy B (2018), and Gopalakrishnan V (2018) may make important contributions to this. In 2015, the average annual citations were highest, which may be related to fewer publications and higher quality of papers. From 2018 to 2021, the average annual citations showed a downward trend, which was contrary to the annual Np, and may be related to the delayed citation of recently published papers.

**Table 1:** The top 10 global cited papers based on total citations in GM/CI.

Paper	DOI	TC	TC per year	Normalized TC
Routy B, 2018, Science	10.1126/science.aan3706	2104	420.80	15.50
Gopalakrishnan V, 2018, Science	10.1126/science.aan4236	1816	363.20	13.37
Sivan A, 2015, Science	10.1126/science.aac4255	1730	216.25	3.83
Vetizou M, 2015, Science	10.1126/science.aad1329	1596	199.50	3.53
Iida N, 2013, Science	10.1126/science.1240527	1116	111.60	2.76
Havel JJ, 2019, Nat Rev Cancer	10.1038/s41568-019-0116-x	956	239.00	16.62
Johnson Ch, 2016, Nat Rev Mol Cell Bio	10.1038/nrm.2016.25	909	129.86	4.34
Honda K, 2016, Nature	10.1038/nature18848	820	117.14	3.91
Pitt JM, 2016, Immunity	10.1016/j.immuni.2016.06.001	538	76.86	2.57
Cabrita R, 2020, Nature	10.1038/s41586-019-1914-8	524	174.67	20.06

### Primary journals

**Figure 3:**
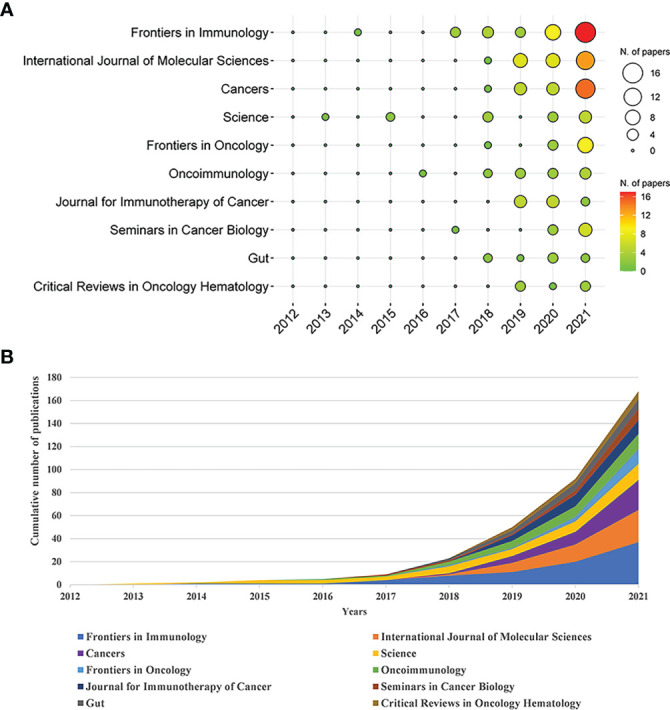
**(A)** The top 10 journals’ annual publications over time in GM/CI (the size of the circle represents the number of publications, and the larger the circle, the more the number of publications; the depth of the circle represents the average annual citation, and the darker the color, the more citations). **(B)** The cumulative number of publications of the top 10 journals in GM/CI.

These papers were published in 295 journals. **Table 2** shows the top 10 journals in the Np. It can be seen that *Frontiers in Immunology* had the most Np (n = 37, 5.56%), followed by *International Journal of Molecular Sciences* (n = 28, 4.21%), and *Cancers* (n = 26, 3.91%). **Figure 3A** depicts annual publications of the top 10 journals over time in GM/CI. **Figure 3B** summarizes the annual changes in the cumulative Np published in the top 10 journals. The cumulative Np published in these journals was 175, accounting for about 26.32% of all papers, indicating that these journals published most of the papers in GM/CI. The TC can show the importance of the journals and H-Index can evaluate the academic influence of journals. **Table 3** shows the top 10 most cited journals, of which *Science* was the most cited, followed by *Nature*, *Nature Reviews Cancer*, *Immunity* and *Annals of Oncology*. *Frontiers in Immunology* has the highest H-index, followed by *Science* and *International Journal of Molecular Sciences*.

**Table 2:** Distribution of top 10 productive journals in GM/CI.

Rank	Journals	Np	IF (2020)	Partition (2020)	Countries
1	Frontiers in Immunology	37	7.561	Q1	Switzerland
2	International Journal of Molecular Sciences	28	5.924	Q1	Switzerland
3	Cancers	26	6.639	Q1	Switzerland
4	Science	14	47.728	Q1	USA
5	Frontiers in Oncology	13	6.244	Q2	Switzerland
6	Oncoimmunology	13	8.110	Q1	USA
7	Journal for Immunotherapy of Cancer	12	13.751	Q1	England
8	Seminars in Cancer Biology	10	15.707	Q1	England
9	Gut	8	23.059	Q1	England
10	Critical Reviews in Oncology Hematology	7	6.312	Q1	USA

**Table 3:** Distribution of top 10 local impact journals in GM/CI.

Rank	Journals	TC	Journals	H-Index
1	Science	9885	Frontiers in Immunology	15
2	Nature	1863	Science	14
3	Nature Reviews Cancer	1476	International Journal of Molecular Sciences	11
4	Immunity	1051	Cancers	10
5	Annals of Oncology	974	Gut	8
6	Nature Reviews Molecular Cell Biology	909	Journal for Immunotherapy of Cancer	8
7	Frontiers In Immunology	762	Oncoimmunology	7
8	Nature Medicine	660	Cancer Immunology Research	6
9	Nature Communications	586	Critical Reviews in Oncology Hematology	6
10	Cancers	570	Seminars in Cancer Biology	6

### Main authors

**Figure 4:**
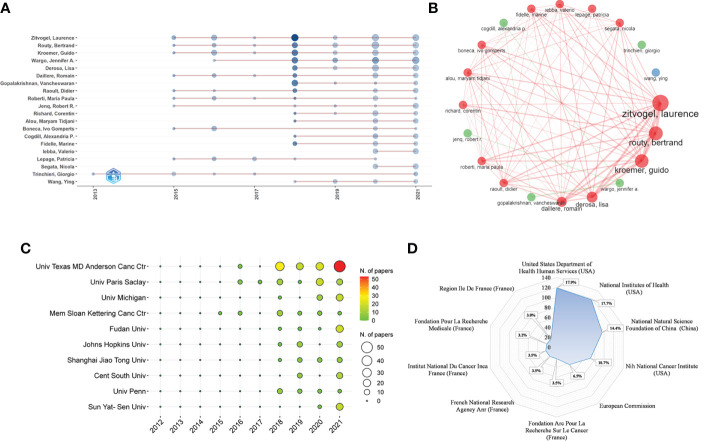
**(A)** The top 20 authors’ annual publication over time in GM/CI (the size of the circle represents the number of publications, and the larger the circle, the more the number of publications; the depth of the circle represents the average annual citation, and the darker the color, the more citations). **(B)** The top 20 authors’ co-authorship network in GM/CI (each node represents an author, the size of the node represents the number of published articles, the line represents the collaboration network between authors, and the thickness of the line represents the strength of collaboration). **(C)** The top 10 institutions’ annual publications over time in GM/CI. **(D)** The top 10 related funding agencies for the support of GM/CI research.

The dataset involved more than 4000 authors. To avoid the repetition caused by name abbreviation, we used the authors’ full names and Web of Science Researcher ID to extract and analyze the Np of authors. **Table 4** lists the top 10 authors in the Np and their H-index, TC, affiliates and countries. As we can see, the top 10 authors were mainly from Univ Texas MD Anderson Canc Ctr in the United States and Gustave Roussy and Univ Paris Saclay in France. Zitvogel L (29/4.36%, 20), Routy B (24/3.61%, 16), Kroemer G (22/3.31%, 16), Wargo JA (22/3.31%, 18), and Derosa L (16/2.41%, 12) ranked the top five in the Np/percentage and H-index. Zitvogel L had the most Np (29), the highest H-index (20) and the most TC (8216), indicating that his papers were of high quality and had a great impact on GM/CI research. **Figure 4A** shows the change in the Np of the top 20 authors over time. Most authors started publishing related papers in 2015 and published the most papers in 2018 (darkest in the graph). **Figure 4B** shows the collaborations of the top 20 authors, we can see that Zitvogel L, Routy B, Kroemer G, Derosa L and Daillere R from Gustave Roussy in France had the closest cooperative relationship in GM/CI.

**Table 4:** The top 10 productive authors in GM/CI.

Rank	Authors	Np	H-index	TC	Affiliations	Countries
1	Zitvogel, Laurence	29	20	8216	Gustave Roussy, Univ Paris Saclay	France
2	Routy, Bertrand	24	16	5999	Gustave Roussy, Univ Paris Saclay	France
3	Kroemer, Guido	22	16	6070	Gustave Roussy, Univ Paris	France
4	Wargo, Jennifer A.	22	18	5153	Univ Texas MD Anderson Canc Ctr	USA
5	Derosa, Lisa	16	12	2977	Gustave Roussy, Univ Paris Saclay	France
6	Daillere, Romain	13	11	5243	Gustave Roussy, Univ Paris Saclay	France
7	Gopalakrishnan, Vancheswaran	9	9	3553	Univ Texas MD Anderson Canc Ctr	USA
8	Raoult, Didier	9	8	4596	Aix Marseille Univ	France
9	Roberti, Maria Paula	9	7	4862	Gustave Roussy, Univ Paris Saclay	France
10	Jenq, Robert R.	8	6	796	Univ Texas MD Anderson Canc Ctr	USA

### Major institutions and countries/regions

More than 1200 institutions were involved in this study, of which the top 10 most productive institutions were shown in **Table 5**. Among them, Univ Texas MD Anderson Canc Ctr, Univ Paris Saclay, Univ Michigan, Mem Sloan Kettering Canc Ctr and Fudan Univ were the top five. The distribution of the top 10 institutions was as follows: six institutions in the United States, three institutions in China and one institution in France. **Figure 4C** depicts the annual Np of the top 10 institutions over time. Among them, Mem Sloan Kettering Canc Ctr was the institution that started earlier (2015), while Univ Texas MD Anderson Canc Ctr published the most papers. **Figure 4D** depicts the main funding agencies, mainly from the US, China and France, indicating these countries have strong support for GM/CI research.

**Table 5:** The top 10 productive countries/regions and institutions involved in GM/CI.

Rank	Countries	Np	TC	AC	Institutions	Np
1	USA	176	13691	77.79	Univ Texas MD Anderson Canc Ctr (USA)	124
2	China	168	3322	19.77	Univ Paris Saclay (France)	51
3	Italy	57	1275	22.37	Univ Michigan (USA)	29
4	France	38	6510	171.32	Mem Sloan Kettering Canc Ctr (USA)	28
5	Japan	32	1889	59.03	Fudan Univ (China)	25
6	Canada	23	1453	63.17	Johns Hopkins Univ (USA)	22
7	Australia	19	245	12.89	Shanghai Jiao Tong Univ (China)	22
8	Spain	17	251	14.76	Cent South Univ (China)	20
9	United Kingdom	14	875	62.50	Univ Penn (USA)	20
10	Germany	12	376	31.33	Sun Yat-Sen Univ (China)	20

**Figure 5:**
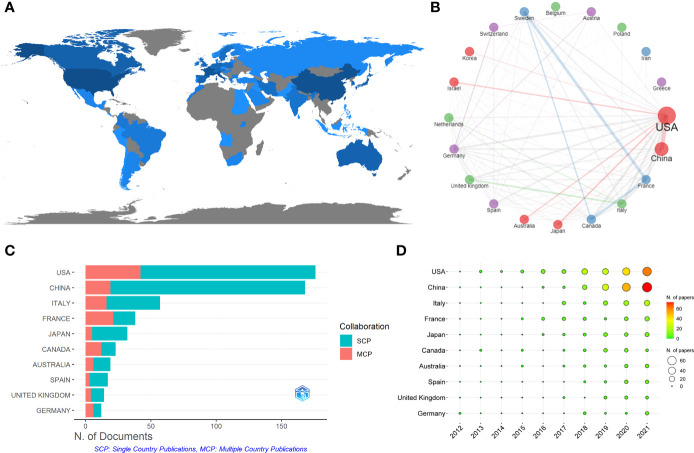
**(A)** Distribution of publications from different countries/regions in GM/CI. **(B)** International collaboration network of the top 20 countries in GM/CI. **(C)** The top 10 countries’ papers partnerships in GM/CI. **(D)** The top 10 countries’ annual publications over time in GM/CI.

These papers were from 43 countries (**Figure 5A**). **Table 5** shows that the papers were mainly published in the United States (176) and China (168), accounting for about 52% of the total output. Analysis of national cooperation in the top 20 countries was carried out through the collaboration network (**Figure 5B**). We can see that the United States was at the center of international cooperation and had the closest cooperation with China. A total of 42 papers in the United States came from international cooperation, followed by China with 19 papers (**Figure 5C**). More than half of the papers of France and Canada come from international cooperation in the top 10 countries. From the time distribution of papers (**Figure 5D**), before 2019, the United States ranked first overall in the annual Np. Since 2019, China’s annual publications had exceeded the United States.

### Analysis of highly cited papers in GM/CI research

#### Historical cited papers of GM/CI research

**Figure 6:**
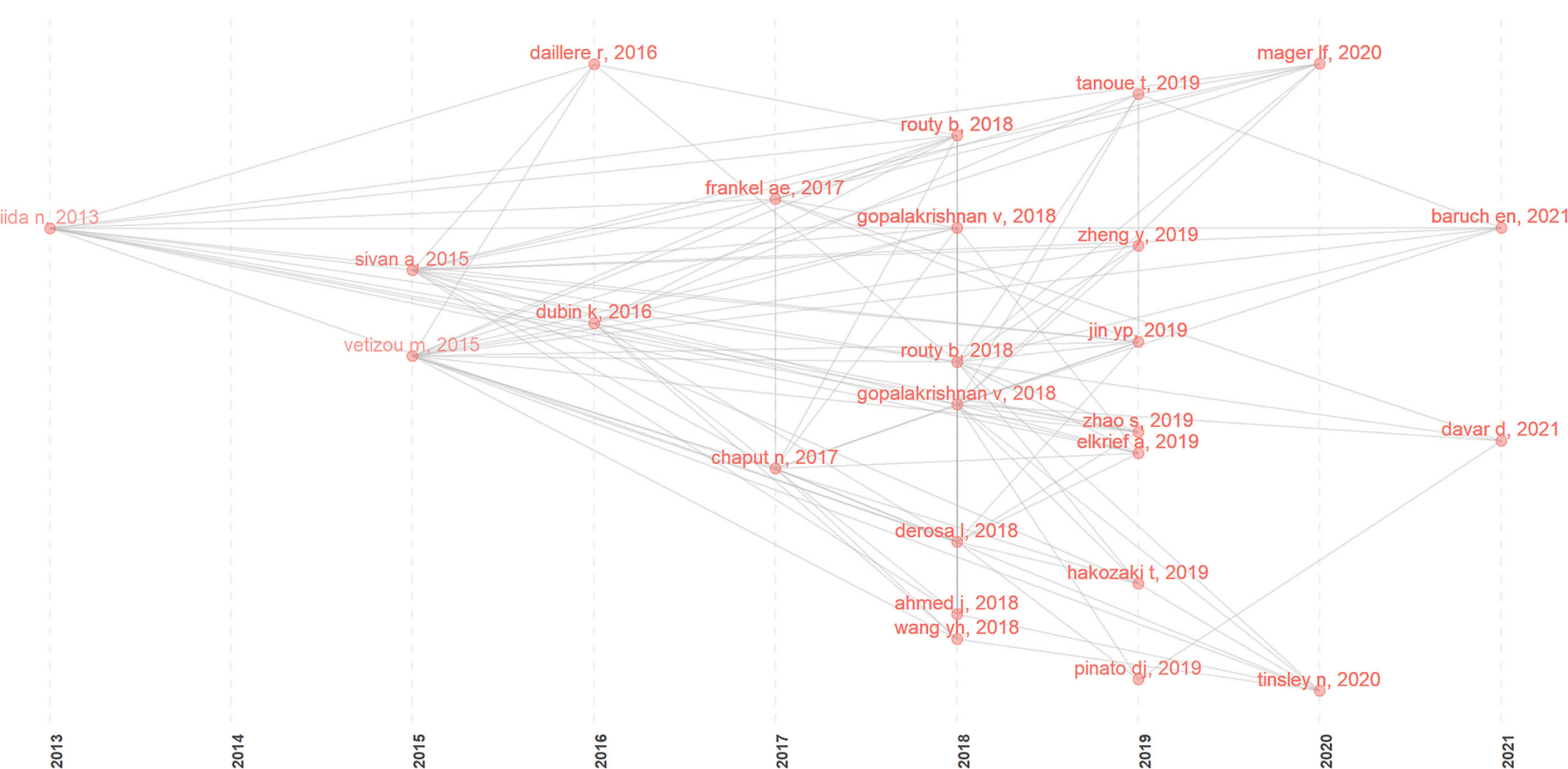
Historical direct citation network in GM/CI research (the gray lines between the dots indicate the citation relationship, and each dot represents an article, distinguished by author name and published year).

Using the historical cited paper visualization analysis in the Bibliometrix of R package, we found some classic papers in GM/CI (**Figure 6**). To examine the quality of research contents of the classic papers, two metrics, LCS and GCS, were used. LCS is for local citation score, and it corresponds to the number of citations of an article in the downloaded dataset. GCS is for global citation score, which represents the times an article has been cited by all documents in the WoS database (**Table 6**).

**Table 6:** The papers of historical direct citation network in the GM/CI.

No.	Title	Document type	Journals	First author	Year	LCS	GCS
1	Commensal bacteria control cancer response to therapy by modulating the tumor microenvironment	Animal study	Science	Iida, N.	2013	70	1116
2	Commensal Bifidobacterium promotes antitumor immunity and facilitates anti-PD-L1 efficacy	Animal study	Science	Sivan, A.	2015	155	1730
3	Anticancer immunotherapy by CTLA-4 blockade relies on the gut microbiota	Animal study	Science	Vetizou, M.	2015	148	1596
4	Enterococcus hirae and Barnesiella intestinihominis Facilitate Cyclophosphamide-Induced Therapeutic Immunomodulatory Effects	Animal and clinical study	Immunity	Daillere, R.	2016	30	347
5	Intestinal microbiome analyses identify melanoma patients at risk for checkpoint-blockade-induced colitis	Clinical study	Nat. Commun.	Dubin, K.	2016	39	496
6	Metagenomic Shotgun Sequencing and Unbiased Metabolomic Profiling Identify Specific Human Gut Microbiota and Metabolites Associated with Immune Checkpoint Therapy Efficacy in Melanoma Patients	Clinical study	Neoplasia	Frankel, AE.	2017	40	261
7	Baseline gut microbiota predicts clinical response and colitis in metastatic melanoma patients treated with ipilimumab	Clinical study	Ann. Oncol.	Chaput, N.	2017	64	514
8	Gut microbiome influences efficacy of PD-1-based immunotherapy against epithelial tumors	Animal and clinical study	Science	Routy, B.	2018	184	2104
9	Gut microbiome modulates response to anti-PD-1 immunotherapy in melanoma patients	Animal and clinical study	Science	Gopalakrishnan, V.	2018	172	1816
10	The gut microbiota influences anticancer immunosurveillance and general health	Review	Nat. Rev. Clin. Oncol.	Routy, B.	2018	25	211
11	The Influence of the Gut Microbiome on Cancer, Immunity, and Cancer Immunotherapy	Review	Cancer Cell	Gopalakrishnan, V.	2018	33	459
12	Negative association of antibiotics on clinical activity of immune checkpoint inhibitors in patients with advanced renal cell and non-small-cell lung cancer	Clinical study	Ann. Oncol.	Derosa, L.	2018	58	376
13	Use of broad-spectrum antibiotics impacts outcome in patients treated with immune checkpoint inhibitors	Clinical study	Oncoimmunology	Ahmed, J.	2018	17	72
14	Fecal microbiota transplantation for refractory immune checkpoint inhibitor-associated colitis	Clinical study	Nat. Med.	Wang, YH.	2018	18	276
15	A defined commensal consortium elicits CD8 T cells and anti-cancer immunity	Animal study	Nature	Tanoue, T.	2019	44	396
16	Gut microbiome affects the response to anti-PD-1 immunotherapy in patients with hepatocellular carcinoma	Clinical study	J. Immunother. Cancer	Zheng, Y.	2019	13	129
17	The Diversity of Gut Microbiome is Associated With Favorable Responses to Anti-Programmed Death 1 Immunotherapy in Chinese Patients With NSCLC	Clinical study	J. Thorac. Oncol.	Jin, YP.	2019	25	144
18	Antibiotics are associated with attenuated efficacy of anti-PD-1/PD-L1 therapies in Chinese patients with advanced non-small cell lung cancer	Clinical study	Lung Cancer	Zhao, S.	2019	17	73
19	Antibiotics are associated with decreased progression-free survival of advanced melanoma patients treated with immune checkpoint inhibitors	Clinical study	Oncoimmunology	Elkrief, A.	2019	17	91
20	Impact of prior antibiotic use on the efficacy of nivolumab for non-small cell lung cancer	Clinical study	Oncol. Lett.	Hakozaki, T.	2019	15	60
21	Association of Prior Antibiotic Treatment With Survival and Response to Immune Checkpoint Inhibitor Therapy in Patients With Cancer	Clinical study	Jama Oncol.	Pinato, DJ.	2019	31	189
22	Microbiome-derived inosine modulates response to checkpoint inhibitor immunotherapy	Animal study	Science	Mager, LF.	2020	13	212
23	Cumulative Antibiotic Use Significantly Decreases Efficacy of Checkpoint Inhibitors in Patients with Advanced Cancer	Clinical study	Oncologist	Tinsley, N.	2020	14	65
24	Fecal microbiota transplant promotes response in immunotherapy-refractory melanoma patients	Clinical study	Science	Baruch, EN.	2021	15	226
25	Fecal microbiota transplant overcomes resistance to anti-PD-1 therapy in melanoma patients	Clinical study	Science	Davar, D.	2021	15	190

Several classic articles appeared from 2013 to 2021 (**Figure 6** clearly shows the citation relationship), most of which were published in 2018 and 2019. As we can see, the earliest node was a paper in 2013, which found that disruption of the commensal microbiota could impair subcutaneous tumor response to CpG-oligonucleotide immunotherapy and platinum chemotherapy (24). In 2015, two studies (25, 26) in *Science* showed that the GM can influence the efficacy of ICIs, and manipulating the GM may modulate ICIs. In 2016, a prospective study demonstrated that increased representation of bacteria belonging to the Bacteroidetes phylum is correlated with resistance to the development of ICI-induced colitis (27). Another study showed that *Enterococcus hirae* and *Barnesiella intestinihominis* can promote anti-tumor immune response (28). In 2017, an article showed that baseline GM enriched with *Faecalibacterium* and other Firmicutes were associated with a beneficial response to ipilimumab and a higher incidence of ipilimumab-induced colitis (29). A clinical study identified specific human GM and metabolites associated with the efficacy of ICIs in melanoma patients (30).

In 2018, two studies (31, 32) published in *Science* revealed that primary resistance to ICIs can be attributed to GM, significant differences were observed in the diversity and composition of the GM between responders and non-responders, antibiotics and fecal microbiota transplantation (FMT) can influence the anti-tumor effect of ICIs. Two clinical studies (33, 34) showed that antibiotic use is associated with the reduced clinical benefit of ICIs in cancer patients. Simultaneously, a clinical study (35) showed that FMT can treat refractory ICIs-associated colitis. Two reviews (12, 36) further summarized the impact of the GM on CI. In 2019, Tanoue T et al. (37) identified 11 healthy human-associated bacterial strains that work together to induce interferon-γ-producing CD8 T cells, which in turn, together with ICIs, effectively inhibit tumor growth. Two clinical studies (38, 39) revealed a strong correlation between GM diversity and response to anti-PD-1 immunotherapy in hepatocellular carcinoma and non-small cell lung cancer (NSCLC) patients. Four clinical studies (40–43) found that antibiotic therapy is associated with reduced response to ICIs in routine practice and affects the prognosis of patients treated with ICIs. In 2020, an article (44) showed that microbiome-derived inosine can modulate response to ICIs immunotherapy. In 2021, two clinical studies (45, 46) showed that FMT can promote melanoma patients’ response to CI and overcome resistance to anti-PD-1 therapy.

#### Top 15 most cited papers in GM/CI research

Highly cited articles are generally articles with extremely high academic value and great professional influence in a field, which are also one of the most valuable indicators in bibliometric methods. **Table 7** and **Table 8** list the 15 most cited papers in original research and reviews worldwide.

**Table 7:** The top 15 cited original research related to the GM/CI.

Rank	Title	First author	Year	Journals	IF	Partition	TC
1	Gut microbiome influences efficacy of PD-1-based immunotherapy against epithelial tumors	Routy, B.	2018	Science	47.728	Q1	2104
2	Gut microbiome modulates response to anti-PD-1 immunotherapy in melanoma patients	Gopalakrishnan, V.	2018	Science	47.728	Q1	1816
3	Commensal Bifidobacterium promotes antitumor immunity and facilitates anti-PD-L1 efficacy	Sivan, A.	2015	Science	47.728	Q1	1730
4	Anticancer immunotherapy by CTLA-4 blockade relies on the gut microbiota	Vetizou, M.	2015	Science	47.728	Q1	1596
5	Commensal Bacteria Control Cancer Response to Therapy by Modulating the Tumor Microenvironment	Iida, N.	2013	Science	47.728	Q1	1116
6	Baseline gut microbiota predicts clinical response and colitis in metastatic melanoma patients treated with ipilimumab	Chaput, N.	2017	Ann. Oncol.	32.976	Q1	514
7	Intestinal microbiome analyses identify melanoma patients at risk for checkpoint-blockade-induced colitis	Dubin, K.	2016	Nat. Commun.	14.919	Q1	496
8	A defined commensal consortium elicits CD8 T cells and anti-cancer immunity	Tanoue, T.	2019	Nature	49.962	Q1	396
9	Tumor Microbiome Diversity and Composition Influence Pancreatic Cancer Outcomes	Riquelme, E.	2019	Cell	41.584	Q1	377
10	Negative association of antibiotics on clinical activity of immune checkpoint inhibitors in patients with advanced renal cell and non-small-cell lung cancer	Derosa, L.	2018	Ann. Oncol.	32.976	Q1	376
11	Enterococcus hirae and Barnesiella intestinihominis Facilitate Cyclophosphamide-Induced Therapeutic Immunomodulatory Effects	Daillere, R.	2016	Immunity	31.745	Q1	347
12	Fecal microbiota transplantation for refractory immune checkpoint inhibitor-associated colitis	Wang, Y.	2018	Nat. Med.	53.44	Q1	276
13	Metagenomic Shotgun Sequencing and Unbiased Metabolomic Profiling Identify Specific Human Gut Microbiota and Metabolites Associated with Immune Checkpoint Therapy Efficacy in Melanoma Patients	Frankel, AE.	2017	Neoplasia	5.715	Q2	261
14	Fecal microbiota transplant promotes response in immunotherapy-refractory melanoma patients	Baruch, EN.	2021	Science	47.728	Q1	226
15	Microbiome-derived inosine modulates response to checkpoint inhibitor immunotherapy	Mager, LF	2020	Science	47.728	Q1	212

**Table 8:** The top 15 cited reviews related to the GM/CI.

Rank	Title	First author	Year	Journals	IF	Partition	TC
1	The evolving landscape of biomarkers for checkpoint inhibitor immunotherapy	Havel, JJ.	2019	Nat. Rev. Cancer	60.716	Q1	956
2	The microbiota in adaptive immune homeostasis and disease	Honda, K.	2016	Nature	49.962	Q1	820
3	Resistance Mechanisms to Immune-Checkpoint Blockade in Cancer: Tumor-Intrinsic and -Extrinsic Factors	Pitt, JM.	2016	Immunity	31.745	Q1	538
4	The Influence of the Gut Microbiome on Cancer, Immunity, and Cancer Immunotherapy	Gopalakrishnan, V.	2018	Cancer Cell	31.743	Q1	459
5	Microbiota: a key orchestrator of cancer therapy	Roy, S.	2017	Nat. Rev. Cancer	60.716	Q1	414
6	Gut microbiota modulation of chemotherapy efficacy and toxicity	Alexander, JL.	2017	Nat. Rev. Gastroenterol. Hepatol.	46.802	Q1	359
7	The microbiome, cancer, and cancer therapy	Helmink, BA.	2019	Nat. Med.	53.44	Q1	341
8	The microbiome in cancer immunotherapy: Diagnostic tools and therapeutic strategies	Zitvogel, L.	2018	Science	47.728	Q1	308
9	Biomarkers for predicting efficacy of PD-1/PD-L1 inhibitors	Yi, M.	2018	Mol. Cancer	27.401	Q1	292
10	The hallmarks of successful anticancer immunotherapy	Galluzzi, L.	2018	Sci. Transl. Med.	17.992	Q1	267
11	The Role of the Microbiome in Cancer Development and Therapy	Bhatt, AP.	2017	CA-Cancer J. Clin.	508.702	Q1	244
12	Anticancer effects of the microbiome and its products	Zitvogel, L	2017	Nat Rev Microbiol.	60.633	Q1	218
13	The gut microbiota influences anticancer immunosurveillance and general health	Routy, B.	2018	Nat. Rev. Clin. Oncol.	66.675	Q1	211
14	Gut Microbiota and Cancer: From Pathogenesis to Therapy	Vivarelli, S.	2019	Cancers	6.639	Q1	203
15	Biomarkers for Clinical Benefit of immune Checkpoint inhibitor Treatment-A Review From the Melanoma Perspective and Beyond	Buder-Bakhaya K.	2018	Front. Immunol.	7.561	Q1	136

The top 15 most cited original articles were mainly published between 2013 and 2021, nearly half of which were from the *Science*, with an average IF of 39.828. Some original research has been outlined above, including the efficacy of ICIs targeting cytotoxic T lymphocyte-associated antigen 4 (CTLA-4) (26), PD-1 (31, 32) and PD-L1 (25) were closely related to GM; the GM can predict clinical response and adverse effects in cancer patients treated with ICIs (27, 29, 30); antibiotics can alter the GM and affect the clinical efficacy of ICIs (33); specific gut bacteria (28, 37), microbial metabolites such as inosine (44) and probiotics such as *Bifidobacterium* (25) can enhance host resistance and the efficacy of ICIs; FMT can play an active role in altering the GM to modulate CI, which can not only promote response in patients with immunotherapy-refractory melanoma (45), but also successfully treat refractory ICI-related colitis (35). Aside from that, the GM has a regulatory effect on the tumor microenvironment. Related studies showed that the GM can mediate its effects by modulating myeloid-derived cell function in the tumor microenvironment (24), and the interactions of the pancreatic adenocarcinoma microbiota and the GM can influence the host immune response and tumor growth (47).

A review is an informative document condensed on the basis of a large number of collected papers, which can provide researchers with comprehensive information, guide scientific research, help readers understand the new progress, existing problems and future directions of the discipline, and provide timely guidance for further research on the basis of grasping the dynamics of the discipline. The top 15 most cited review articles were mainly published between 2016 and 2019, nearly half of their articles were from *Nature* and its sub-journals, with an average IF of 71.897. Among them, four review articles (48–51) mentioned GM can be used as a biomarker for predicting the efficacy of ICIs immunotherapy. Several review articles (12, 13, 24, 36, 52–56) outlined the effect of the GM on oncology, immunity, and the efficacy and adverse effects of CI. There is a crosstalk between GM and immune cells. A paper (57) outlined the interactions of T and B cells of the immune system and the GM. A review (58) mentioned interventions to modify the microbiome to aid in cancer treatment, including FMT, antibiotic therapy, prebiotics, dietary interventions, or drugs that alter the composition of the GM.

Given that high-cited papers were mostly published in the high IF-journals and were mainly original research, we aggregated GM/CI-related original research articles published in high IF-journals (may get more citations in the future) and ranked them by TC (**Supplementary Material S2**). A total of 20 articles were extracted, of which *Science* had the most publications (n=12), accounting for 60%, *Nature* and *Cell* each published three papers, each accounting for 15%, and *Nature Medicine* published two papers, accounting for 10%. Compared with previous studies (24–26, 31, 32), which mainly focused on the relationship between GM and CI outcomes, the mechanism research of the GM/CI correlation from the perspective of metabolism (44, 59) and immunity (60–62) and the intervention research that alter the GM to improve the efficacy of CI, such as FMT (45, 46), dietary fiber and probiotics (63), Enterococcus peptidoglycan (64) and Enterococcus bacteriophage (65), have gradually become the focus of GM/CI research in high-IF journals in the last few years.

### Analysis of keywords

#### Analysis of high-frequency keywords in GM/CI

**Figure 7:**
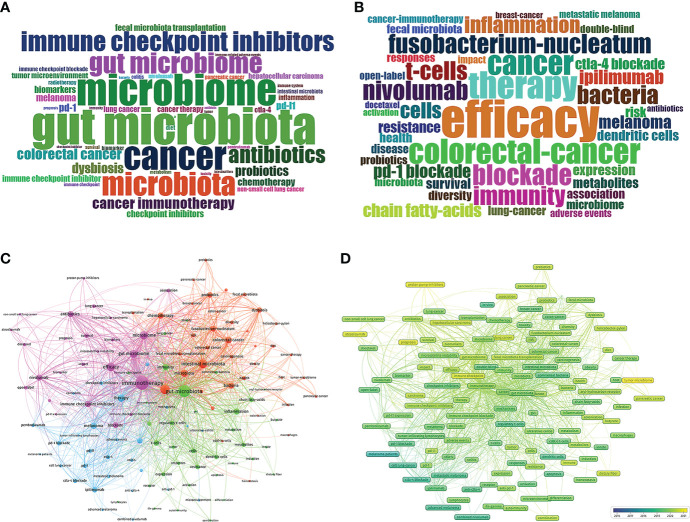
**(A)** Distribution of top 50 Author Keywords in GM/CI. **(B)** Distribution of top 50 Keywords Plus in GM/CI. **(C)** Cluster analysis of high-frequency keywords (frequency ≥10) based on all keywords of publications in GM/CI (different colors represent different clusters, the size of the circle represents the frequency the keywords appear, and the thickness of the line represents the total link strength between keywords). **(D)** Trends in keywords (frequency ≥ 10) over time based on all keywords of publications in GM/CI (the blue boxes represent the earliest keywords and the yellow boxes represent the latest keywords).

To determine the hotspots in GM/CI, it is necessary to examine an important index in the literature—keywords. The frequency of keywords and the cluster analysis of high-frequency keywords based on the co-occurrence of keywords can indicate the current research hotspots and themes in a certain field. In this study, a total of 2447 keywords (1109 author’s keywords and 1138 keywords plus) were extracted from the imported papers. The author’s keywords and keywords plus with the top 50 frequencies were represented in wordcloud *via* the R tool’s Bibliometrix packages (**Figure 7A, B**). Among author’s keywords, the most used keywords (exclude search terms) are “efficacy”, “antibiotics”, “probiotics”, “biomarkers”, “fecal microbiota transplantation”, “diet”, etc. Among keywords plus, the most used keywords (exclude search terms) are “*Fusobacterium-nucleatum*”, “inflammation”, “regulatory t-cells”, “short chain fatty-acids”, “resistance”, “dendritic cells”, “survival”, “metabolites”, etc.

Based on the number of publications with co-occurrence keywords and the association strength of keywords, a clustering analysis was performed to assess the relationship between the identified items *via* VOSviewer. This study treated each clustered keywords as a category and classifies all individuals based on the same color. As shown in **Figure 7C**, we divided common keywords (frequency ≥ 10, 127 items) into 4 clusters based on the total link strength between keywords and represented them with different colors.

**Cluster 1** (purple topic, 25 items) is mainly related to the association between GM and efficacy of CI (such as GM as a biomarker to predict the efficacy and prognosis of CI) and the effect of some drugs such as antibiotics on efficacy of CI. **Cluster 2** (red topic, 39 items) is primarily related to the links between GM and cancer (such as *Fusobacterium-nucleatum* and colorectal-cancer), as well as the important role of diet, FMT, probiotics and prebiotics in cancer and CI. **Cluster 3** (green topic, 35 items) is mainly related to the mechanism by which the GM affects cancer and immunotherapy, including immunity (such as regulatory T cells and dendritic cells), inflammation, and metabolism (such as short chain fatty acids and butyrate). **Cluster 4** (blue topic, 28 items) is related to specific applications of ICIs in melanoma and adverse events such as colitis. The previous research on the links between GM and CI mainly focused on melanoma may be related to ICIs were the first approved for melanoma treatment.

#### Analysis of the development trends of high-frequency keywords in GM/CI

The development and change of keywords over time can reflect the evolution of frontier knowledge to a certain extent and lead the future research direction in a field. Through the Overlay Visualization of VOSviewer, similar to concurrency graphs, we predicted the trends for the next few years in GM/CI. VOSviewer uses different colors for each keyword in the image based on the average year they appear and frequency (year. frequency) in all included publications, as shown in **Figure 7D**, where the blue boxes represent the earliest keywords and the yellow boxes represent keywords appear in recent years and may be future research directions. From 2016 to 2021, there were relatively unbalanced development dynamics in four clusters, more yellow nodes were in the cluster 1 and 2 and the main keywords for yellow nodes included “tumor microbiome (2020.93)”, “proton pump inhibitors (2020.69)”, “prognosis (2020.64)”, “atezolizumab (2020.55)”, “immune checkpoint” (2020.50), “immune checkpoint inhibitor” (2020.48), “hepatocellular carcinoma” (2020.44), “lung cancer” (2020.44), “pancreatic cancer (2020.33)”, etc.

## Discussion

At present, immunotherapy plays an increasingly important role in tumor treatment, among which ICIs have received the most attention, but there are still some problems that could lead to treatment delay and even early termination, such as low response rate to ICIs and immune-related adverse events in some cancer patient. Over the past decade, with the deepening understanding of GM and the increasing evidence which suggest GM is one of the modulators that can alter the efficacy and toxicity of CI, the studies on improving the efficacy of CI by modulating GM have gradually increased. Based on the above reasons, this paper conducts a bibliometric analysis on the role of GM in CI for the first time and allows researchers to have a general understanding of the current status of the links between GM and CI.

### Analysis of characteristics of literature

The number and trend of publications in a certain field can reflect the development stage it has gone through. From the annual Np, 2012-2014 and before 2012 (only 5 articles in 2004-2011) belonged to the initial stage of GM/CI research, 2015-2017 was in a steady development stage, and 2018-2021 was in a stage of rapid development. In 2010, a human gut microbial gene catalog established by metagenomic sequencing opened up a new direction for the GM research. In 2011, the first ICI Ipilimumab was approved by the US FDA for the second-line treatment of advanced melanoma, marking a new era of CI. Since then, GM and CI began to collide. In 2015, two blockbuster studies (25, 26) found that GM can influence the efficacy of ICIs. Correspondingly, the correlation research between GM and CI began to increase. Since 2018, the GM/CI research has received increasing attention from scholars, which may be due to the rapid development of ICIs and GM research and widespread clinical application of ICIs.

Our analysis showed that *Frontiers in Immunology*, *International Journal of Molecular Sciences* and *Cancers* published the most papers. Moreover, *Frontiers in Immunology* had the highest H-index. *Frontiers in Immunology* is the official journal of the International Union of Immunological Societies (IUIS), supports full open access, and is a leading journal in the field of immunology. The higher the academic quality of an academic journal, the easier it will attract the attention of the academic community and be read by more experts and scholars, and the greater its influence may be. The most cited articles were published in *Science*, followed by *Nature*, *Nature Reviews Cancer*, *Immunity*, and *Annals of Oncology*. These journals are internationally renowned and have greater international influence. Among them, *Science* has the highest TC, and the top 5 highly cited papers were all from the journal, indicating this influential and prestigious journal is more likely to publish high-quality research in the future. *Nature* and its related sub-journals published most of the highly cited reviews, indicating that these journals are more likely to publish high-quality review articles in the future.

Countries, institutions and authors may not affect the quality of papers, but bibliometrics can show their contributions to a particular field. These papers mainly came from the United States and China, followed by Italy, France and Japan. The United States had the most Np and was at the core of global cooperation. China had seen a surge in the Np and had outnumbered the United States in terms of annual Np. This can be related to the high attention and financial support of the government and the community of these two countries on the GM program and CI research. For example, the United States launched the Human Microbiome Project and the National Microbiome Initiative in 2007 and 2016, respectively, and China launched the Chinese Academy of Sciences Microbiome Program in 2017. Furthermore, a 2020 study (66) showed that the United States and China dominated cancer cell therapy in the world and China had surpassed the United States in the number of clinical trials. Notably, despite the Np in China was considerable, the average citations were far lower than that of other countries and the highly cited papers were lacking, suggesting that China still needs to further improve the quality of articles.

The top 10 institutions were from the United States, China and France, half of which were in the United States, demonstrating its good scientific research capabilities in GM/CI. Univ Texas MD Anderson Canc Ctr, one of the three earliest comprehensive cancer treatment centers designated by the National Cancer Program in the United States and one of the most authoritative cancer hospitals in the world, had published the most articles. Most of the top 10 authors were from Gustave Roussy and Univ Paris Saclay in France. The former is the first cancer center in Europe, and the latter is a comprehensive and world-class research university jointly built by a number of French educational institutions and national institutes. The most published author was Laurence Zitvogel, an immunologist from France, who has contributed to the field of CI and is a leading pioneer in the study of the relationship between GM and CI, with the highest H-index and TC. In terms of the impact of papers, Routy B is the first author of the most cited article and Zitvogel L is its corresponding author. In addition, Gopalakrishnan V, Sivan A, Vetizou M and Iida N also participated in the writing of this highest cited paper, and they respectively contributed a high cited paper as the first author. They can be considered as outstanding contributioners.

### Historical evolution in the GM/CI research

Through highly cited papers and historical citation network analysis, we have a general understanding of the development process of GM/CI research. From 2012 to 2014, animal studies (24, 67) showed that GM can influence local and systemic inflammation and stimulate anti-tumor immune responses of CpG-oligonucleotide immunotherapy and chemotherapy. In 2015, two blockbuster preclinical animal studies (25, 26) were the first to link GM to ICIs response. One of them found that the composition of GM can affect the treatment response of PD-L1 and oral administration of *Bifidobacterium* can improve anti-tumor effects of PD-L1 by enhancing the function of dendritic cells (25). The other one (26) showed that the anti-tumor effect of CTLA-4 was inhibited in germ-free or antibiotic-treated melanoma mice, and the supplementation with *Bacteroides fragilis* could overcome this defect, which was related to the activation of Th1 cells in tumor draining lymph node and the induction of maturation of DCs in tumors. Since then, more and more research efforts have been devoted to GM/CI studies. In 2018, correlation studies of GM and CI began to shift from preclinical mouse models to cancer patients. Two representative studies (31, 32) showed that composition and diversity of GM can predict response to ICIs immunotherapy, which were of milestone significance in the field of GM/CI. One of the studies with the highest TC clearly demonstrated the significant impact of GM on CI, revealed that primary resistance to ICIs can be attributed to aberrant GM composition, antibiotics inhibited the clinical benefit of ICIs and FMT can improved the anti-tumor response of ICIs, and suggested that regulation of specific gut bacteria can avoid the primary resistance of ICIs (31). The other study (32) found that in melanoma patients receiving PD-1 immunotherapy, significant differences were observed in the diversity and composition of the GM between responders and non-responders. Systemic anti-tumor immunity was enhanced in responder patients and germ-free mice with favorable GM that received FMT from responders. From 2019 to 2020, many clinical studies began to appear, some retrospective clinical studies (40–43) have shown that antibiotics are associated with decreased survival and reduced response to ICIs in cancer patients. Some prospective clinical studies (38, 39) confirmed a significant association between GM and ICIs outcomes in advanced solid tumors. Simultaneously, mechanistic studies are also further in-depth to microbial metabolites. In 2021, clinical research went further into the therapeutic area. Two clinical trials (45, 46) showed that FMT from ICIs responders combined with anti-PD-1 therapy can overcome the resistance to PD-1 in melanoma patients.

In addition, as can be seen from the International Clinical Trials Registry Platform (ICTRP, https://trialsearch.who.int/), the GM/CI-related clinical observational trials (ChiCTR2100047045, ChiCTR2100047044, NCT04682327, NCT05008861, NCT05065515, JPRN-UMIN000041822) and interventional trials especially FMT-related studies (ChiCTR2100043472, NCT04163289, NCT04758507, NCT04924374, NCT05273255, NCT05279677, NCT05286294) have also gradually increased in recent years. All in all, the GM/CI research has gone through the process from basic research to clinical application. Notably, since 2020, the COVID-19 pandemic has challenged the status of immunotherapy (68) and had an important impact on GM/CI research. A study (69) conducted in the early stages of the pandemic showed that scientists were spending significantly less time on research than they did before the pandemic and scientific output for many researchers has declined in 2020 compared to 2019. Moreover, affected by the COVID-19 pandemic, some researchers have also begun to link GM/CI research with the COVID-19 (70–72), for example, immunocompromised cancer patients, including those with COVID-19, may restore their anti-tumor immune responses when receiving ICIs therapy (71), COVID-19 may affect the GM, and then could affect the efficacy of immunotherapy (70).

### Research hotspots and trends of GM/CI

Hotspots are determined by high-frequency keywords, cluster analysis of keywords and highly cited papers. By analyzing these elements, this study found that the current hotspots of GM/CI research are mainly concentrated in three aspects: (1) GM can be used as a biomarker to predict the efficacy and toxicity of CI. (2) GM can affect the efficacy of CI and some potential mechanisms may explain this phenomenon. (3) Modulation of GM can improve or reduce the efficacy of CI.

Firstly, currently available immunotherapy drugs are expensive and only some cancer patients respond to CI, requiring the identification of reliable predictive biomarkers to improve the accuracy of treatment selection. Some articles (48–51) and a meta-analysis (73) have mentioned that GM can be used as a biomarker to distinguish whether patients will respond to ICIs or not and predict the efficacy and adverse reaction of CI. In general, the higher the diversity of GM, the higher the response to CI (32). A 2017 study (29) showed that baseline GM may predict clinical response and colitis in patients with metastatic melanoma treated with ipilimumab. A review (74) summarizing the relationship between the GM and immunotherapy of different cancers indicated that the GM can be used as a predictive biomarker for clinical response in CI. Some bacterial species such as *Akkermansia muciniphila*, *Bacteroides fragilis*, *Bifidobacterium* spp., *Faecalibacterium* spp., *Ruminococcaceae* spp., *Eubacterium limosum* and *Enterococcus hirae* have been associated with favorable anti-tumor immune response (12, 39, 75). Moreover, a prospective study (27) demonstrated that increased abundance of the Bacteroidetes phylum is correlated with resistance to the development of ICIs-induced colitis. Another study (76) showed that the toxicity of ICIs was associated with a significant increase in the abundance of *Bacteroides intestinalis*.

Secondly, the efficacy is the most critical factor to evaluate the anti-tumor treatment, and it is also the most concerned part of doctors and patients. Therefore, most studies on the correlation between the GM and CI mainly focus on the effect of GM on CI outcomes. It has been confirmed that the GM has a significant relationship with CI outcomes in melanoma (77), NSCLC (78), hepatocellular carcinoma (39) and renal cell carcinoma (79). Many studies (25, 26, 31) have shown that the GM can affect the host’s response to PD-1, PD-L1 and CTLA-4, and the supplementation of specific bacterial species can restore or enhance the response to ICIs (75). Furthermore, the GM is also associated with response and toxicity of chimeric antigen receptor (CAR) T-cells therapy (80). As far as the current studies are concerned, the GM has an important impact on CI mainly by affecting immunity and metabolism. On the one hand, the effect of GM on CI acts through the local and systemic immune systems and the production of anti-inflammatory cytokines (13, 75). T and B cells of the immune system interact with the GM to influence CI (57). The GM plays a role in inducing the formation or reprogramming of immune cells such as regulatory T cells (81), interferon-γ-producing CD8 T cells (37) and interferon-dependent monocyte (61), which are associated with anti-tumor response. On the other hand, the GM can impact local and systemic anti-tumor immune responses by means of metabolites, such as inosine (44) and short-chain fatty acids (82), which may be the link between GM and CI efficacy. Moreover, the GM can modulate the outcomes of ICIs through antigen-specific mechanisms and antigen-independent mechanisms (83). The immuno-oncology-microbiome axis, also known as cancer-microbiome-immune axis, has been proposed to explain this phenomenon (84, 85).

Thirdly, the GM is a potential target for enhanced efficacy and reduced toxicity of CI. Modifying GM can not only improve clinical efficacy of CI, but also reduce the occurrence of adverse events. Microbiota-centric interventions could be the next breakthrough in CI. At present, the related research on intervening the GM to adjust CI mainly includes the following aspects: (1) Fecal microbiota transplantation: FMT is a common method for clinically altering GM, which can improve the efficacy of ICIs and reduce their side effects (86). In 2018, a clinical study (35) in *Natural Medicine* demonstrated that FMT can effectively treat ICIs-associated colitis. In 2021, two clinical trials in *Science* found that FMT can promote the response in immunotherapy-refractory melanoma patients (45) and FMT from ICIs-responders combined with anti-PD-1 therapy can overcome resistance to PD-1 blockade in melanoma patients (46). (2) Provision of specific microbiota: Microbiota with immune-enhancing effects or modified beneficial microbiota can be used as adjuvants for immunotherapy. For example, a consortium of 11 bacterial strains isolated from healthy human feces can enhance the efficacy of ICIs (37). Enterococcus peptidoglycan (64) and Enterococcus bacteriophage (65) can also improve curative effect of CI. (3) Probiotics and Prebiotics: Probiotic use is associated with favorable clinical outcomes in patients receiving anti-PD-1 therapy (87). Oral administration of *Bifidobacterium* combined with anti-PD-1 therapy can improve the effect of PD-L1 by enhancing the function of dendritic cells (25) and *Bifidobacterium* can reduce the toxicity of CTLA-4 by relying on regulatory T cells (88). Administration of adjunctive probiotic *Lactobacillus rhamnosus Probio-M9* can enhance the efficacy of anti-PD-1 by restoring antibiotic-disrupted GM (89) and *Lactobacillus rhamnosus GG* can enhance anti-tumor immune responses in a CD8 T cell-dependent manner (90). (4) Diet and Dietary fiber: The interactions between dietary patterns and GM can affect the inflammatory response and the efficacy of CI. For instance, Western diet has negative effects on both the GM and immune system, while the Mediterranean diet is the exact opposite (91); Microbiota modulation with a high-fiber diet can trigger the intratumoral monocyte reprogramming and improve the efficacy of ICIs (61). Furthermore, dietary fiber can also influence GM and anti-tumor immune response (63). Combining dietary and nutritional approaches that alter the GM with immunotherapy could provide a new avenue for cancer therapy (68). Several studies (NCT04645680, NCT04866810, NCT05356182) are investigating the relationship between dietary interventions and ICIs. (5) Antibiotics: Unlike the above, antibiotics mainly play a negative role in CI. Antibiotics can disrupt the homeostasis of GM, thereby affecting the efficacy of CI (31, 33). At present, several clinical studies (40–43) have shown that antibiotic use is associated with reduced response to ICIs and affects the prognosis of patients treated with ICIs. Two meta-analyses also showed that antibiotics use is associated with worse clinical outcomes in cancer patients treated with ICIs (92, 93).

Keywords analysis showed that some emerging research fields (the yellow nodes in **Figure 7D**) are becoming the focus of scholars and may be future research directions. (1) Tumor microbiome (TM): Although the correlation of the GM and CI outcomes has been well studied, data on the role of the local TM, an important part of the tumor microenvironment, are insufficient. Some studies have explored the correlation between the TM and CI and found the TM can also promote oncogenesis (94) and affect the treatment effect (95). The interaction of the TM and the GM influenced the host immune response and tumor growth (47). A newly published article showed that the GM can affect the TM through translocation or other manners (15). (2) Proton pump inhibitors (PPIs): Antacids such as PPIs are often used in cancer patients, and these drugs have the potential to alter GM and the efficacy of anti-tumor therapy (96, 97). Two meta-analyses demonstrated that concomitant antacid could alter the activity of ICIs, PPIs were inversely associated with progression free survival and overall survival (98, 99). (3) Prognosis: By measuring the abundance and diversity of GM, we can quantify the relative proportions of recognized “beneficial” or “harmful” bacteria, which can predict treatment outcomes and prognosis, and help guide treatment decisions and regimens. In the future, the composition of the GM may be combined with other known outcome-related factors to predict the prognosis of CI. (4) Immune checkpoint inhibitors: As shown in the above keywords analysis, ICIs are not only a current research hotspot, but also a research focus in the next few years. ICIs are booming in the field of tumor treatment with good efficacy and safety. In the future, with the wide application of ICIs among different tumor types, there will be increasing papers related to GM and ICIs. (5) Hepatocellular carcinoma, lung cancer and pancreatic cancer: Previous research have focused on melanoma, and as indications for ICIs expand, more cancer types are being studied. For example, some studies show that the GM is associated with clinical response to CI in pancreatic cancer and hepatocellular carcinoma (38, 94, 100), and its composition may predict response to ICIs in patients with these cancers and influence the efficacy of CI (38, 100).

### Limitations of the article

Our research still has some limitations. First, only the publications included in the SCIE of WoSCC were searched and English articles were included, although it represents a certain level of research, it cannot cover all studies in different languages around the world. This is mainly because English is the most widely used language in the world and the WoS is one of the most authoritative citation index databases for global academic information. Moreover, it is difficult to mix documents of different languages and databases for bibliometric analysis simultaneously in the same software. Meanwhile, we also searched other document types of papers (such as editorial material, meeting abstract) and other languages and found that they had few citations and low impact. In view of this, this study makes this choice. Second, bibliometric analysis tools cannot analyze the complete text of publications at present, and some information may be ignored, which can also be used as a shortcoming. Therefore, we analyzed research content of highly cited papers and provide an overview of the mechanisms by which GM and CI interact and how to modulate the GM to enhance the efficacy of CI to make up for the shortcomings and flaws of this paper. Third, this study is only an analysis of papers at the current stage. Even though we have found the top cited articles, newly published articles may also have higher influence but may be cited less currently. With the rapid development of research on the correlation between GM and CI, more papers will be available for analysis.

## Conclusion

This study shows that the research attention of GM/CI has gradually increased in recent 10 years; specific GM can be used as a biomarker to distinguish patients who respond to ICIs, determine the effectiveness of ICIs, and predict the efficacy and toxicity of CI. Modifying GM can not only improve cancer patients’ clinical efficacy but also reduce the occurrence of adverse events, thereby improving the patients’ survival and prognosis. Microbiota-centric interventions can be the next breakthrough in CI. Concretely speaking, we can change the GM of patients who do not respond to CI or drug-resistant patients by diet adjustment, probiotics use, FMT and so on, so as to restore the response of patients to CI and improve their survival. Research trend terms include TM, PPIs, prognosis and ICIs. In addition to finding gut-specific microbial signatures associated with CI, identifying tumor-specific microbial characteristics is also necessary and promising. Understanding the mechanism of interactions between GM and TM, and then modulating TM by regulating GM or directly regulating TM to enhance the efficacy of CI is an attractive research direction. Moreover, relevant clinical research on the role of FMT in CI is still limited to small sample case reports. With the continuous development of FMT-related research, it may have broad application prospects in CI. In the future, more in-depth research is needed to identify specific bacteria that affect CI and transform them for clinical application, so as to predict and improve the efficacy of CI and reduce the incidence of adverse reactions.

In summary, with the help of scientometrics and visual analysis methods, this study preliminarily shows the global research status and trends of interactions between GM and CI, provides researchers in related fields with a clearer understanding of the development process of GM/CI through historical direct citation analysis, and provides references for in-depth research in the GM/CI field by summarizing and forecasting current research hotspots and future development directions.

## Data availability statement

The raw data supporting the conclusions of this article will be made available by the authors, without undue reservation.

## Author contributions

SY: writing-original draft preparation, manuscript, investigation, and figure preparation. SZ: manuscript, investigation, and figure preparation. YY: investigation and figure preparation. LJ: investigation, methodology, supervision. YL: conceptualization, methodology, supervision. All authors contributed to the article and approved the submitted version.

## Funding

This work was supported by the National Natural Science Foundation of China Youth Science Foundation Project (No. 81904003) Personnel Training Program of China-Japan Friendship Hospital Elite Project (No. ZRJY2021-TD05) and Beijing Natural Science Foundation Project (No. 7202184).

## Conflict of interest

The authors declare that the research was conducted in the absence of any commercial or financial relationships that could be construed as a potential conflict of interest.

## Publisher’s note

All claims expressed in this article are solely those of the authors and do not necessarily represent those of their affiliated organizations, or those of the publisher, the editors and the reviewers. Any product that may be evaluated in this article, or claim that may be made by its manufacturer, is not guaranteed or endorsed by the publisher.
