# Supplementary material for: Global research trends on the links between gut microbiota and cancer immunotherapy: A bibliometric analysis (2012-2021)
Source: Front Immunol. 2022 Aug 24;13:952546. doi: 10.3389/fimmu.2022.952546 (PMC9449151; doi:10.3389/fimmu.2022.952546)
Supplement: Supplementary file 1 [file DataSheet_1.docx]

**Search strategy**：

TS= (“Gut Microbio*” OR “Gut Microflora” OR “Gut Flora” OR “Gut Microbial Flora” OR “Intestinal Microbio*” OR “Intestinal Microflora” OR “Intestinal Flora” OR “Intestinal Microbial Flora” OR “Gastrointestinal Microbio*” OR “Gastrointestinal Microflora” OR “Gastrointestinal Flora” OR “Gastrointestinal Microbial Flora” OR “Gastrointestinal Microbial Communit*” OR “Fecal Microbio*” OR “Fecal Microflora” OR “Fecal Flora” OR “Fecal Microbial Flora” OR “Faecal Microbio*” OR “Faecal Microflora” OR “Faecal Flora” OR “Faecal Microbial Flora” OR “Gut Bacteri*” OR “Intestinal Bacteri*” OR “Gastrointestinal Bacteri*” OR “Fecal Bacteri*” OR “Faecal Bacteri*” OR “Enteric Bacteri*”) AND TS= (“Immunotherap*” OR “Immune Checkpoint Inhibit*” OR “Immune Checkpoint Block*” OR “PD-L1 Inhibit*” OR“PD-L1 Block*” OR “PD L1 Inhibit*” OR“PD L1 Block*” OR “Programmed Death-Ligand 1 Inhibit*” OR “Programmed Death Ligand 1 Inhibit*” OR “PD-1 Inhibit*” OR “PD-1 Block*” OR “PD 1 Inhibit*” OR “PD 1 Block*” OR “Programmed Cell Death Protein 1 Inhibit*” OR “Programmed Cell Death Protein 1 Block*” OR “PD-1-PD-L1 Block*” OR “PD-1-PD-L1 Inhibit*” OR “PD 1 PD L1 Block*” OR “PD 1 PD L1 Inhibit*” OR “CTLA-4 Inhibit*” OR “CTLA-4 Block*” OR “CTLA 4 Inhibit*” OR “CTLA 4 Block*” OR “Cytotoxic T-Lymphocyte-Associated Protein 4 Inhibit*” OR “Cytotoxic T-Lymphocyte-Associated Protein 4 Block*” OR “Cytotoxic T Lymphocyte Associated Protein 4 Inhibit*” OR “Cytotoxic T Lymphocyte Associated Protein 4 Block*” OR “Checkpoint Inhibit*” OR “Checkpoint Block*” OR “PD-L1” OR “PD-1” OR “CTLA-4” OR “PD L1” OR “PD 1” OR “CTLA 4”) AND TS= (“Tumor*” OR “Cancer*” OR “Neoplasia*” OR “Neoplasm*” OR “Malignanc*” OR “Carcinoma*” OR “Melanoma*” OR “Scrcoma*”)

**Query link:**

https://www.webofscience.com/wos/woscc/summary/d373c2f6-1123-4e11-8bd0-20a73e345fc2-4193b34e/times-cited-descending/1

(The results of this search may change slightly due to updates of the WoS database.)
