# Supplementary material for: Global research trends on the links between gut microbiota and cancer immunotherapy: A bibliometric analysis (2012-2021)
Source: Front Immunol. 2022 Aug 24;13:952546. doi: 10.3389/fimmu.2022.952546 (PMC9449151; doi:10.3389/fimmu.2022.952546)
Supplement: Supplementary file 2 [file Table_1.docx]

**Additional TABLE.** The original research papers related to the GM/CI in the top high-impact factor (IF＞40) journals.

| **Rank** | **Title** | **DOI** | **Year** | **Journals** | **IF** | **Partition** | **TC** |
| --- | --- | --- | --- | --- | --- | --- | --- |
| 1 | Gut microbiome influences efficacy of PD-1-based immunotherapy against epithelial tumors | 10.1126/science.aan3706 | 2018 | Science | 47.728 | Q1 | 2104 |
| 2 | Gut microbiome modulates response to anti-PD-1 immunotherapy in melanoma patients | 10.1126/science.aan4236 | 2018 | Science | 47.728 | Q1 | 1816 |
| 3 | Commensal Bifidobacterium promotes antitumor immunity and facilitates anti-PD-L1 efficacy | 10.1126/science.aac4255 | 2015 | Science | 47.728 | Q1 | 1730 |
| 4 | Anticancer immunotherapy by CTLA-4 blockade relies on the gut microbiota | 10.1126/science.aad1329 | 2015 | Science | 47.728 | Q1 | 1596 |
| 5 | Commensal Bacteria Control Cancer Response to Therapy by Modulating the Tumor Microenvironment | 10.1126/science.1240527 | 2013 | Science | 47.728 | Q1 | 1116 |
| 6 | A defined commensal consortium elicits CD8 T cells and anti-cancer immunity | 10.1038/s41586-019-0878-z | 2019 | Nature | 49.962 | Q1 | 396 |
| 7 | Tumor Microbiome Diversity and Composition Influence Pancreatic Cancer Outcomes | 10.1016/j.cell.2019.07.008 | 2019 | Cell | 41.584 | Q1 | 377 |
| 8 | The human tumor microbiome is composed of tumor type-specific intracellular bacteria | 10.1126/science.aay9189 | 2020 | Science | 47.728 | Q1 | 359 |
| 9 | Fecal microbiota transplantation for refractory immune checkpoint inhibitor-associated colitis | 10.1038/s41591-018-0238-9 | 2018 | Nat. Med. | 53.440 | Q1 | 276 |
| 10 | Fecal microbiota transplant promotes response in immunotherapy-refractory melanoma patients | 10.1126/science.abb5920 | 2021 | Science | 47.728 | Q1 | 226 |
| 11 | Microbiome-derived inosine modulates response to checkpoint inhibitor immunotherapy | 10.1126/science.abc3421 | 2020 | Science | 47.728 | Q1 | 212 |
| 12 | Fecal microbiota transplant overcomes resistance to anti-PD-1 therapy in melanoma patients | 10.1126/science.abf3363 | 2021 | Science | 47.728 | Q1 | 190 |
| 13 | The gut microbiota is associated with immune cell dynamics in humans | 10.1038/s41586-020-2971-8 | 2020 | Nature | 49.962 | Q1 | 91 |
| 14 | Cross-reactivity between tumor MHC class I-restricted antigens and an enterococcal bacteriophage | 10.1126/science.aax0701 | 2020 | Science | 47.728 | Q1 | 82 |
| 15 | Gut microbiota signatures are associated with toxicity to combined CTLA-4 and PD-1 blockade | 10.1038/s41591-021-01406-6 | 2021 | Nat. Med. | 53.440 | Q1 | 43 |
| 16 | Dietary fiber and probiotics influence the gut microbiome and melanoma immunotherapy response | 10.1126/science.aaz7015 | 2021 | Science | 47.728 | Q1 | 33 |
| 17 | A metabolomics pipeline for the mechanistic interrogation of the gut microbiome | 10.1038/s41586-021-03707-9 | 2021 | Nature | 49.962 | Q1 | 32 |
| 18 | Microbiota triggers STING-type I IFN-dependent monocyte reprogramming of the tumor microenvironment | 10.1016/j.cell.2021.09.019 | 2021 | Cell | 41.584 | Q1 | 31 |
| 19 | Enterococcus peptidoglycan remodeling promotes checkpoint inhibitor cancer immunotherapy | 10.1126/science.abc9113 | 2021 | Science | 47.728 | Q1 | 23 |
| 20 | Dysregulation of ILC3s unleashes progression and immunotherapy resistance in colon cancer | 10.1016/j.cell.2021.07.029 | 2021 | Cell | 41.584 | Q1 | 19 |
